# DNA barcoding of marine rocky reef fishes from northern Peru suggests a parapatric speciation in the Tropical Eastern Pacific

**DOI:** 10.1002/ece3.70125

**Published:** 2025-03-05

**Authors:** E. Zavala, R. Britzke, Z. Siccha‐Ramírez, J. L. Ramirez

**Affiliations:** ^1^ Facultad de Ciencias Biológicas Universidad Nacional Mayor de San Marcos Lima Peru; ^2^ Departamento de Ictiología, Museo de Historia Natural Universidad Nacional Mayor de san Marcos Lima Peru

**Keywords:** DNA barcoding, genetic distances, rocky reefs, species delimitation

## Abstract

Northern Peru marks the end of an extensive coastal marine region: The Panama province, which is characterized by predominantly tropical and equatorial features and is home to the only rocky reefs known in Peruvian territory. This unique ecosystem could explain the presence of a diverse range of fish species. However, due to the difficulty of sampling and accessing reef areas, our knowledge of this biodiversity is incomplete. To address this issue, we used DNA barcoding for the study of the fish biodiversity and revealed patterns that may have influenced their evolution throughout the Tropical Eastern Pacific (TEP). A fragment of Cytochrome oxidase subunit I (COI) of 177 samples of rocky reef fishes was sequenced. Intra and interspecific K2P distances were calculated and three species delimitation methods (GMYC, PTP, and bPTP) were used to obtain MOTUs. Both analyses support the conformation of additional MOTUs in samples of *Mugil cephalus*, *Ophichthus zophochir*, *Malacoctenus tetranemus*, *Ariopsis seemanni* and *Halichoeres dispilus*, species with a divergence above 2%. By comparing these sequences with public data, our analysis revealed the existence of COI lineages and suggested potential ecological parapatric speciation in the TEP. More studies using other markers and different approaches are required to confirm the existence of species complexes that could be related to the presence of cryptic species.

## Graphical abstract

Using a DNA barcoding technique we found evidence that reinforce the hypotheses of how parapatric speciation could drive the origin of new species in the TEP.

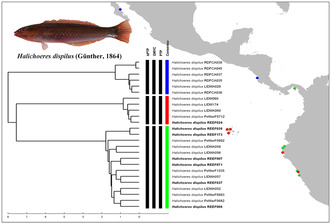

## 1 INTRODUCTION

Northern Peru marks the end of an extensive coastal marine region known as Panama Province, which harbors most of the 70% percent of the Peruvian marine biodiversity (Willenz et al., 2010). This region is also recognized as the southern limit of the Tropical Eastern Pacific (TEP), a marine biogeographic region that extends from the Gulf of California (México) to Cabo Blanco (Piura) (Robertson & Allen, 2015; Robertson & Cramer, 2009). Hooker (2009) has identified this region as a natural limit of the tropical fauna. However, during intense summers, an event known as El Niño and Southern Oscillation (ENSO) can increase the occurrence of tropical species in southern refuge locations such as Isla Foca and Sechura Bay (Piura) (Hooker, 2009) due to the mixture of tropical and equatorial water masses and the existence of a transition zone (Hooker et al., 2013); between the TEP and Temperate South America (TSA) marine realm (Spalding et al., 2007).

The marine ichthyofauna from Peru has been documented in several publications highlighting the Fish identification key of Chirichigno and Vélez (1998) and the “*Commented catalog of marine fish from Peru*” of Chirichigno and Cornejo (2001). Other works such as Hooker (1990, 1993, 2000), Vildoso et al. (1999), Hooker (2009), Britzke et al. (2021) and Siccha‐Ramirez et al. (2022) have focused on the Northern coast of Peru. Many of the fish species treated in these documents have a wide distribution in the Eastern Pacific and can reach the Gulf of California. In fact, about 59% of the fishes listed by Robertson and Allen (2015) in the TEP are vagrant species from temperate provinces (Californian and Peruvian), and at least one‐third of the fishes from the TEP are localized in northern Peru; mainly in rocky coastal and mangrove ecosystems (Robertson & Allen, 2015; Tarazona et al., 2003).

Coral reefs are among the most diverse habitats in marine systems worldwide (Hubert et al., 2012). Reef fishes, which have undergone many adaptive radiation events, are an important fraction that contributes to the ocean biodiversity (Westneat & Alfaro, 2005). However, there aren't coral reefs areas reported for the northern coast of Peru. Instead, rocky reef ecosystems have been described between Cabo Blanco and Punta Sal (Hooker & Ubillus, 2011). Also, the presence of submarine canyons and the Máncora Bank in front of this coast can allow the establishment of benthopelagic communities of tropical origin (SERNANP, 2018).

Rocky reefs are intertidal ecosystems that occur at the interface of the sea and due to marine erosion and the combined action of waves and tides in areas with low sediment input (Sanchez et al., 2015). These ecosystems are important for their support of high marine biodiversity, which is due to the variety of microhabitats such as pools, caves, crevices and canyons. Rocky reefs also provide food and protection in coastal areas against storms, strong waves and sediment transport (Msangameno, 2016). They also act as refuge and breeding areas (Msangameno, 2016).

According to Jiménez (1999), most research on reef fish communities has been conducted in the Caribbean, Australia and the Red Sea. In the TEP, the California Gulf is considered a hotspot for marine biodiversity due to the high concentration of rocky reef ecosystems and their topographic complexity (Aburto & Balart, 2001; Sanchez et al., 2015). However, compared to these regions, reef research in Peru is still insufficient. Although rocky reef fishes have been described on the northern coast of Peru, much of their biodiversity remains undocumented due to the difficulties in sampling and accessing these areas (Vildoso et al., 1999).

Additionally, many of these fish exhibit phenotypic plasticity (Hooker, 2009), represented by complex color patterns that can vary according to the age or sex of the species (Westneat & Alfaro, 2005) which further complicated their identification. Furthermore, given that the TEP represents a dynamic coastal environment characterized by a complex system of oceanic processes, segregated rocky habitats (Palmerín‐Serrano et al., 2021), and the absence of effective barriers to dispersal, Robertson and Cramer (2009) hypothesized parapatric speciation is the main driver for the origin of new species within the TEP. In fact, Rocha and Bowen (2008) recognize that parapatric speciation may be the predominant mode of speciation among reef fishes. Parapatric speciation occurs when new species evolve in contiguous, yet spatially segregated habitats, maintaining contact zones and not completely ceasing gene exchange (Hollocher, 2013), that can lead to the origin of species that are morphologically indistinguishable but genetically different (Cryptic species, Bickford et al., 2007).

In this context, the DNA barcoding approach (Hebert et al., 2003) represents a valuable tool which has been successfully applied in the identification of both cryptic fish species (Jacobina et al., 2020) and fish species with highly sexual dimorphism (Sarhan et al., 2019), common in reef ecosystems. Thus, the purpose of this study is to use the DNA Barcoding approach to molecularly characterize the tropical fish fauna that inhabits or is associated with tropical rocky reefs in northern Peru. As these species are widely distributed in the TEP, genetic analyses can reveal patterns (such as parapatric speciation) that have influenced their evolution.

## 2 MATERIALS AND METHODS

### 2.1 Sampling

**FIGURE 1:**
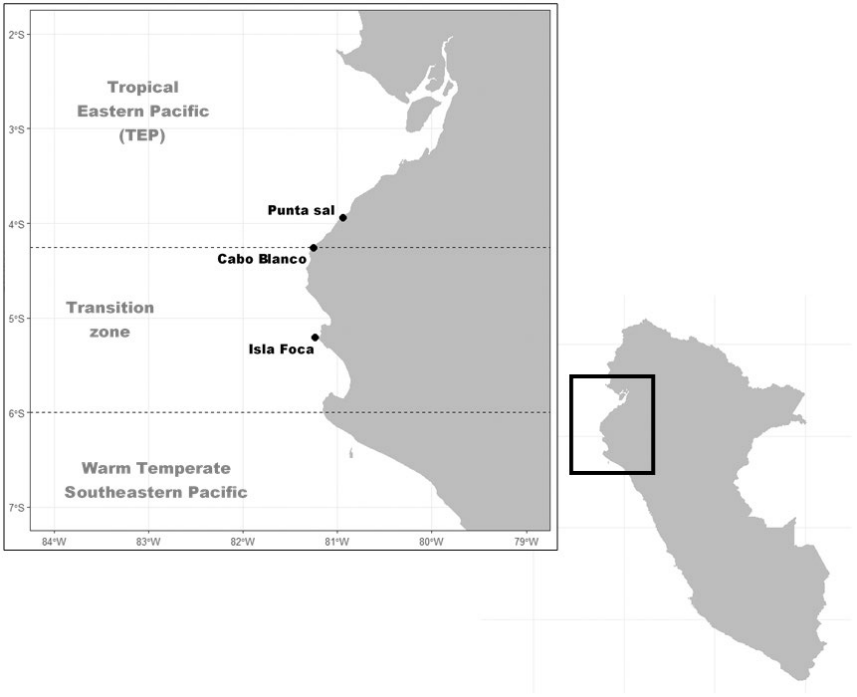
Map of sampling localities. The limits of the TEP and its subdivision were defined by sensu Robertson and Cramer (2009) and the TSA realm is defined by sensu Spalding et al. (2007). A Transition zone between them was defined according Hooker et al. (2013).

We obtained permission to collect specimens from the Ministry of Production under the following resolution number 00432‐2020‐PRODUCE/DGPCHDI. Fish specimens were sampled by scuba diving in rocky reef ecosystems from three areas within the National Reserve “Mar Tropical del Peru” (SERNANP, 2018): Isla Foca (Transition zone), the coastal line between Cabo Blanco – Los Órganos and Arrecifes de Punta Sal in Tumbes (TEP) (Figure 1). A piece of fish tissue muscle (1 cm^3^ approx.) was taken and kept in 96° ethanol. The voucher specimens were photographed and preserved following the recommendations of Lambarri and Espinosa (2018).

### 2.2 DNA isolation, amplification and sequencing

Total DNA isolation from muscle tissues was performed using the Promega Wizard SV Genomic kit; following the manufacturer's instructions. A fragment of 652–655 base pairs of the COI gene was amplified via PCR using the primers Fish F1‐R1 5′ and Fish F2‐R2 described by Ward et al. (2005). The PCR mix had a final volume of 15 μL including 0.25 mM of each dNTP, 1× of Taq buffer, 1.8 mM of MgCl_2_, 0.4 M of each primer and 0.5 U Taq DNA polymerase.

The PCR cycles were performed in a thermo cycler BIO‐RAD T100. The PCR consisted of the following steps: an initial denaturalization for 5 min at 92°C, followed by 35 cycles of denaturalization at 92°C (45 s), hybridization at 45–55°C for 45 s, elongation at 72°C for 45 s and a final extension for 10 min at 72°C. Amplified fragments were visualized in Agarose gel at 1% with Buffer TAE 1× and stained by SYBR Safe DNA gel stain. Positive PCR reactions were purified with the AmpliClean™ Cleanup kit and sent to Macrogen Korea to be sequenced.

The electropherograms were visualized, assembled and edited in the softwares Geneious v.4.8.5 (https://www.geneious.com/) and CodonCode v.9.0.2 (https://www.codoncode.com/). Conting sequences were uploaded to the BOLD Systems platform (Ratnasingham & Hebert, 2007) under two projects: REEF ‘*Los arrecifes rocosos del norte de Perú: Estudiando y conservando la ictiofauna a través de ADN barcode*’, and PMFSH ‘*Fishes biodiversity from Peru*’ where barcodes were generated and uploaded to GenBank under accession PP390264 – PP390495.

### 2.3 Phylogenetic and distance analysis

Sequences were aligned using the ClustalW (Thompson et al., 1997), integrated into the software BioEdit v.7.0.5.3 (Hall, 1999). Initial clustering of individuals was performed using the Neighbor‐Joining (NJ) method based on the Kimura 2‐parameters (K2P) substitution model (Kimura, 1980), using 1000 *bootstrap* replications in the software MEGA X (Kumar et al., 2018). Intra and interspecific distances were calculated using SPdel (Ramirez et al., 2023); where the maximum intraspecific distance, minimum interspecific distance and their corresponding nearest neighbor were computed.

A Bayesian Inference tree was constructed using the HKY + I + G model (Hasegawa et al., 1985) selected in jModelTest v.2.1.10 (Darriba et al., 2012). The tree was built using a Relaxed Clock Log Normal and the Birth‐Death model in the software BEAST v2.6.2 (Bouckaert et al., 2019) in the CIPRES website platform (Miller et al., 2010), based on 300 million replicates with sampling at every 60,000 trees. The convergence of Markov chains was revised in the program Tracer v1.7.1 (Rambaut et al., 2018), where all the values of Effective Sample Size (ESS) were above 200. Trees were summarized into a single target tree in the program TreeAnnotator v.2.6.2 (Bouckaert et al., 2019) using a 10% of burn‐in, where the posterior probability was used as a measure support of the branches. The obtained tree was visualized in FigTree v1.4.3 (Rambaut & Drummond, 2009).

### 2.4 Species delimitation methods

Three algorithms were applied to perform the species delimitation: The Generalized Mixed Yule Coalescent (GMYC) (Fujisawa & Barraclough, 2013), the Poisson Tree Process (PTP) and the Bayesian Poisson Tree Process (bPTP) (Zhang et al., 2013) in the SPdel pipeline (Ramirez et al., 2023); using the Bayesian tree as an input. The congruence among the delimitation of Molecular Taxonomic Units (MOTUs) was evaluated by comparing the clusters inferred from each algorithm to obtain a consensus. For species that resulted in the formation of additional MOTUs, we performed a separate species delimitation analysis along with sequences from the project PMFSH “Fishes biodiversity from Peru” (individuals from localities out of our study area) and public databases, following the same methodology as described above (See Table S2).

## 3 RESULTS

### 3.1 Sampling

In total, 226 fishes from the rocky reef and associated areas were collected, where 82 nominal species were morphologically identified corresponding to individuals from 36 different families (See Table S1). All 82 nominal species were used in the genetic distance analysis.

### 3.2 Genetic distances analysis

**FIGURE 2:**
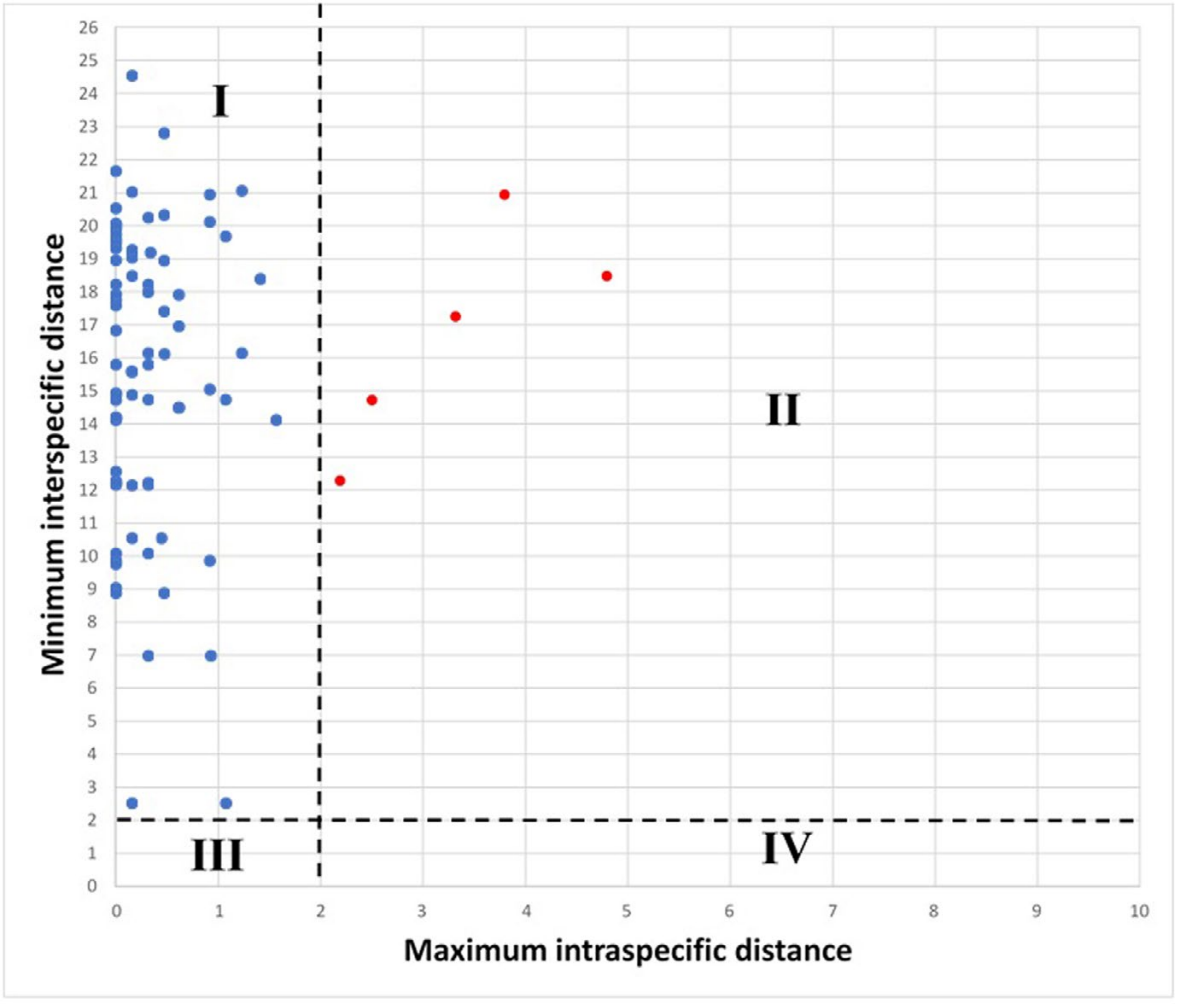
Intraspecific distances compared to interspecific COI K2P distances for individual species of marine rocky reef fishes from northern Peru. Red dots are samples that surpassed the 2% threshold.

Genetic distances based on the K2P substitution model are presented in Table 1. To interpret these results, the values of maximum intraspecific distances (*x*‐axis) and minimum interspecific distances (*y*‐axis) were plotted, with a conservative threshold of 2% (Hebert et al., 2003) dividing the graph into four quadrants (see Figure 2). According Hebert et al. (2004), these quadrants are defined as: (I) intraspecific distance <2%; interspecific distance >2%: concordant with current taxonomy, (II) intraspecific distance >2%; interspecific distance >2%: probably different species, candidate to taxonomic division, (III) intraspecific distance <2% and interspecific distance <2%: recent divergence, hybridization or synonym and (IV) intraspecific distance >2% and interspecific distance <2%: probable misidentification of the specimen.

**TABLE 1:** Genetic distances were calculated for marine rocky reef fishes using the K2P model.

Species	Max (%)	NN	DtoNN (%)
*Abudefduf concolor*	1.23938	*Abudefduf troschelii*	16.13811
*Abudefduf troschelii*	0.30769	*Abudefduf concolor*	16.13811
*Alphestes immaculatus*	0.30769	*Alphestes multiguttatus*	6.97572
*Alphestes multiguttatus*	0.92882	*Alphestes immaculatus*	6.97572
*Anisotremus interruptus*	0.30769	*Microlepidotus brevipinnis*	15.78721
*Apogon dovii*	0.92756	*Apogon pacificus*	9.84665
*Apogon pacificus*	0	*Apogon dovii*	9.84665
*Ariopsis seemanni*	**3.31946**	***Hyporthodus acanthistius***	**17.24392**
*Azurina atrilobata*	0.46172	*Azurina intercrusma*	10.5338
*Azurina intercrusma*	0.15361	*Azurina atrilobata*	10.5338
*Balistes polylepis*	0	*Kyphosus vaigiensis*	17.74431
*Bathygobius ramosus*	0.15361	*Kyphosus elegans*	19.17972
*Bodianus diplotaenia*	0.61729	*Paralabrax callaensis*	17.90672
*Brotula clarkae*	0.34745	*Lutjanus argentiventris*	19.17541
*Calamus brachysomus*	0	*Lutjanus argentiventris*	19.59516
*Caulolatilus affinis*	0	*Kyphosus elegans*	19.47534
*Centropomus nigricensis*	0	*Chaetodipterus zonatus*	20.51708
*Cephalopholis panamensis*	0	*Paranthias colonus*	18.94853
*Chaetodipterus zonatus*	0	*Kyphosus vaigiensis*	16.81412
*Chaetodon humeralis*	1.23747	*Anisotremus interruptus*	21.04697
*Cheilodactylus variegatus*	0.92612	*Oplegnathus insignis*	15.03528
*Chromis alta*	0.15361	*Stegastes flavilatus*	15.56521
*Cirrhitus rivulatus*	0	*Oplegnathus insignis*	17.58488
*Coryphopterus urospilus*	0.92756	*Lutjanus argentiventris*	20.9342
*Cratinus agassizii*	0.3074	*Paralabrax callaensis*	12.14344
*Decodon melasma*	0	*Stegastes flavilatus*	19.90848
*Diodon holocanthus*	0.30769	*Diodon hystrix*	10.07371
*Diodon hystrix*	0	*Diodon holocanthus*	10.07371
*Diplectrum conceptione*	0.61729	*Serranus huascarii*	14.48649
*Echiophis brunneus*	0	*Ophichthus zophochir*	12.26515
*Epinephelus labriformis*	0.15798	*Mycteroperca xenarcha*	15.58519
*Gerres simillimus*	0	*Mycteroperca xenarcha*	19.30934
*Gymnothorax equatorialis*	1.55794	*Gymnothorax phalarus*	14.11321
*Gymnothorax phalarus*	0	*Gymnothorax equatorialis*	14.11321
*Halichoeres dispilus*	**2.50603**	***Halichoeres notospilus***	**14.70896**
*Halichoeres notospilus*	0	*Halichoeres dispilus*	14.70896
*Holacanthus passer*	0	*Pomacanthus zonipectus*	18.2088
*Hyporthodus acanthistius*	0.15355	*Alphestes multiguttatus*	12.13022
*Hypsoblennius brevipinnis*	0	*Hypsoblennius paytensis*	20.06015
*Hypsoblennius paytensis*	0.15361	*Abudefduf concolor*	19.02187
*Johnrandallia nigrirostris*	0.92612	*Chaetodipterus zonatus*	20.11488
*Kyphosus elegans*	0.15361	*Kyphosus vaigiensis*	2.51248
*Kyphosus vaigiensis*	1.0814	*Kyphosus elegans*	2.51248
*Labrisomus multiporosus*	0.3074	*Cheilodactylus variegatus*	20.24102
*Lutjanus argentiventris*	0.47469	*Kyphosus elegans*	16.11294
*Lythrypnus dalli*	0.15355	*Ophichthus zophochir*	24.5412
*Malacoctenus tetranemus*	**3.79772**	***Oplegnathus insignis***	**20.92305**
*Microlepidotus brevipinnis*	0	*Anisotremus interruptus*	15.78721
*Mugil cephalus*	**4.79884**	***Mugil setosus***	**18.46677**
*Mugil setosus*	0.15361	*Mugil cephalus*	18.46677
*Mycteroperca xenarcha*	0	*Hyporthodus acanthistius*	12.55363
*Nicholsina denticulata*	1.08531	*Hypsoblennius paytensis*	19.67036
*Ophichthus zophochir*	**2.19471**	***Echiophis brunneus***	**12.26515**
*Ophioblennius steindachneri*	0.15361	*Scartichthys gigas*	14.86463
*Oplegnathus insignis*	0	*Kyphosus elegans*	14.92049
*Paralabrax callaensis*	0	*Cratinus agassizii*	12.14344
*Paranthias colonus*	0.6161	*Lutjanus argentiventris*	16.94607
*Pareques lanfeari*	0	*Pareques viola*	12.2162
*Pareques viola*	0.30769	*Pareques lanfeari*	12.2162
*Plagiotremus azaleus*	0	*Chaetodipterus zonatus*	19.99913
*Pomacanthus zonipectus*	0.3074	*Holacanthus passer*	18.2088
*Prionotus stephanophrys*	0.46225	*Ariopsis seemanni*	20.31569
*Pronotogrammus multifasciatus*	0.46225	*Rypticus nigripinnis*	18.93768
*Pseudobalistes naufragium*	0	*Balistes polylepis*	19.73664
*Rypticus nigripinnis*	0	*Cirrhitus rivulatus*	17.92037
*Scartichthys gigas*	0	*Ophioblennius steindachneri*	14.86463
*Scorpaena histrio*	1.39978	*Kyphosus vaigiensis*	18.37766
*Scorpaena mystes*	0	*Hyporthodus acanthistius*	21.65006
*Scorpaenodes xyris*	0.3096	*Cratinus agassizii*	17.98016
*Serranus huascarii*	0.61652	*Diplectrum conceptione*	14.48649
*Serranus psittacinus*	0.15355	*Paralabrax callaensis*	19.2517
*Sphoeroides annulatus*	1.08531	*Sphoeroides lobatus*	14.73035
*Sphoeroides lobatus*	0.30769	*Sphoeroides annulatus*	14.73035
*Stegastes acapulcoensis*	0	*Stegastes beebei*	9.74302
*Stegastes beebei*	0	*Stegastes rectifraenum*	9.02322
*Stegastes flavilatus*	0.46225	*Stegastes rectifraenum*	8.87237
*Stegastes rectifraenum*	0	*Stegastes flavilatus*	8.87237
*Sympterygia brevicaudata*	0.46225	*Ophichthus zophochir*	22.79201
*Thalassoma lucasanum*	0.46225	*Halichoeres notospilus*	17.39587
*Tomicodon chilensis*	0.15361	*Paralabrax callaensis*	21.01568
*Urobatis tumbesensis*	0	*Urotrygon chilensis*	14.19241
*Urotrygon chilensis*	0	*Urobatis tumbesensis*	14.19241

*Note*: In bold: Values that exceed the 2% Threshold.

Abbreviations: DtoNN, Distance to NN (Minimum interspecific distance); Max, Maximum intraspecific distance; NN, Nearest neighbor.

Thus, a large number of species were located in the quadrant (I); reflecting a concordance with the morphological identification. However, intraspecific distance values above the 2% threshold were found in five species: *Mugil cephalus* (4.79%), *Ariopsis seemanni* (3.32%), *Ophichthus zophochir* (2.19%), *Malacoctenus tetranemus* (3.80%), and *Halichoeres dispilus* (2.51%), which could indicate a possible taxonomic separation among their individuals inhabiting the TEP.

### 3.3 Species delimitation

In the species delimitation, the number of MOTUs did not show significant differences across the methods applied (GMYC: 88, PTP and bPTP: 87) and the number of consensus MOTUs resulted in 87 (See Figures S1 and S2), a quantity that differs from the number of nominal species identified in this study (82 species). The additional MOTUs correspond to individuals of *Mugil cephalus*, *Ophichthus zophochir*, *Malacoctenus tetranemus*, *Ariopsis seemanni*, and *Halichoeres dispilus*. Therefore, species delimitation was performed for each species, except for *A. seemanni*, due to the lack of georeferenced COI sequences in public databases.

**FIGURE 3:**
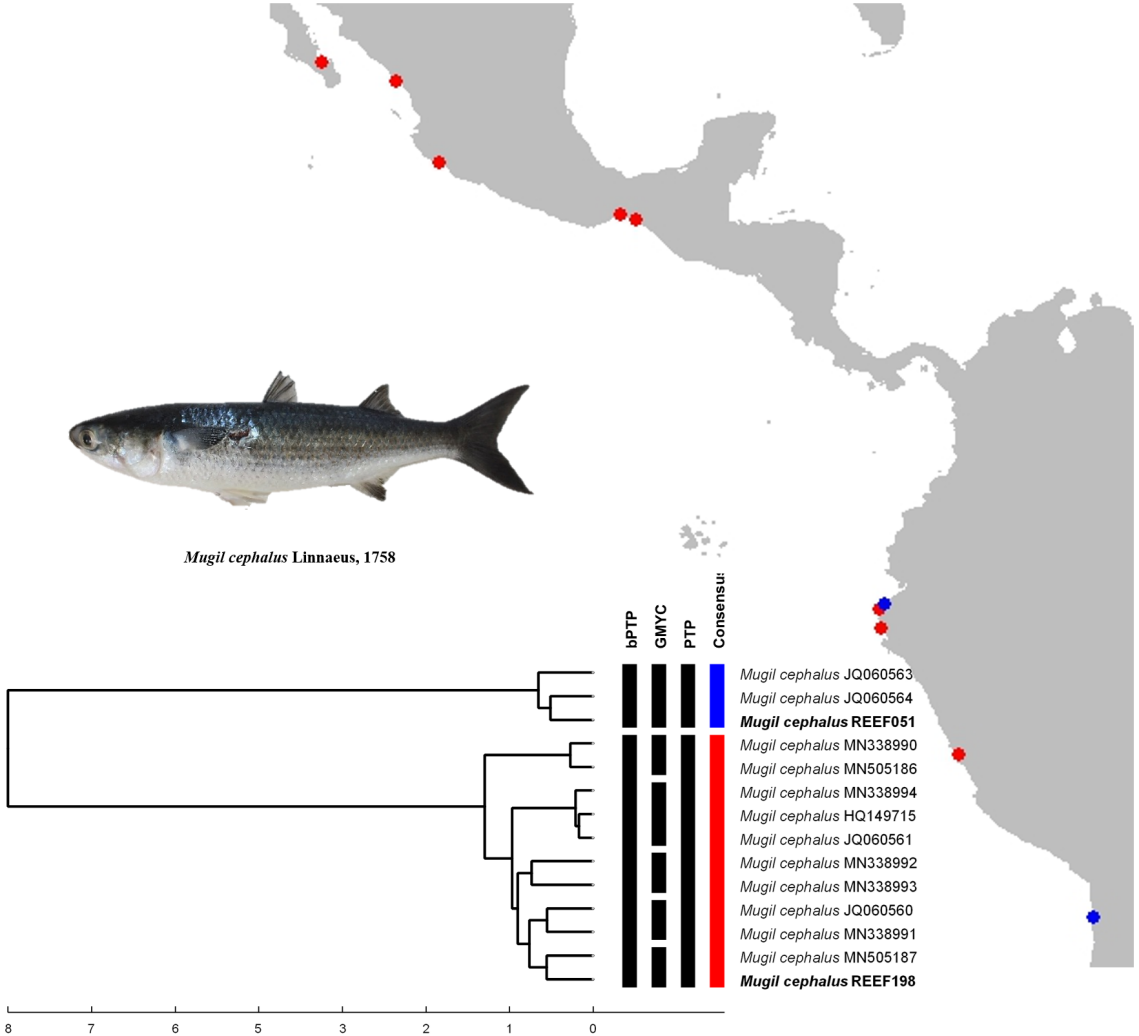
Species delimitation of *M. cephalus* individuals from the EP. Individuals in bold are samples that were used in the main analysis.

**FIGURE 4:**
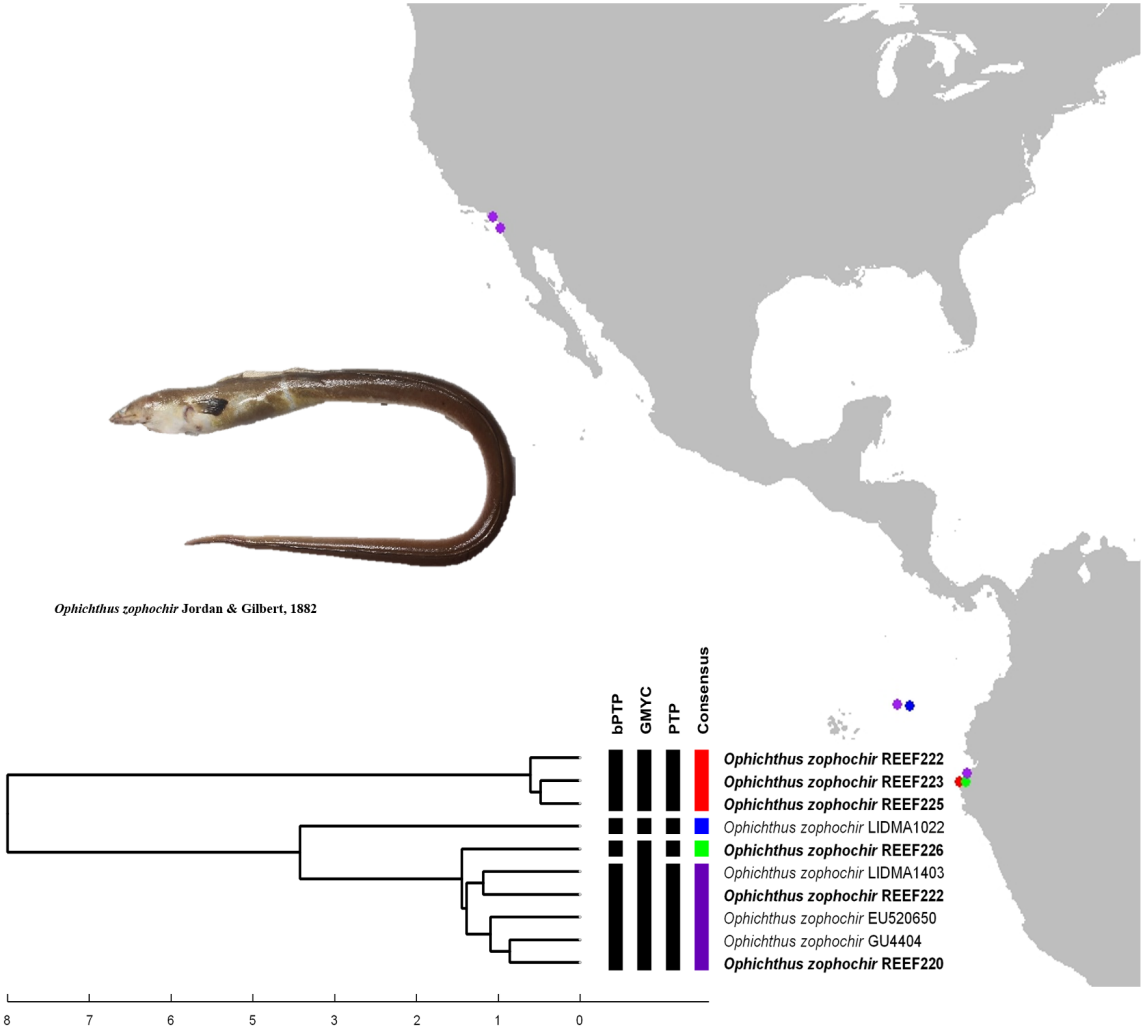
Species delimitation of the *O. zophochir* from the EP. Individuals in bold are samples that were used in the main analysis.

**FIGURE 5:**
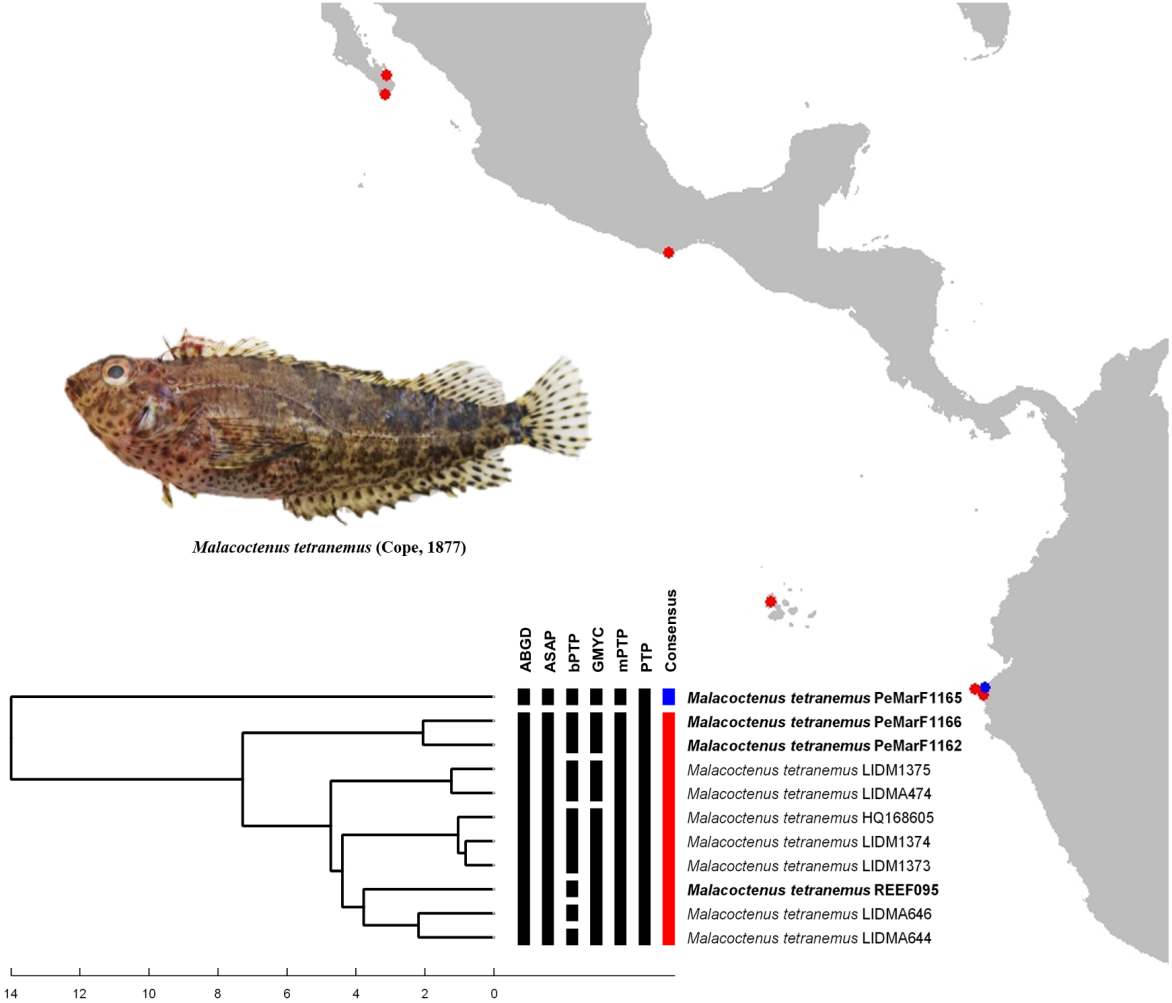
Species delimitation of *M. tetranemus* from the EP. Individuals in bold are samples from northern Peru that were used in the main analysis.

The species delimitation analysis for *Mugil cephalus*, resulted in two MOTUs (Figure 3). The first MOTU comprised individuals from Iquique (northern Chile) and one specimen collected in Cabo Blanco (Piura); while the second MOTU included one specimen from Punta Sal (Tumbes), and individuals from Chiapas, Colima, Mazatlán and La Paz (México). For *Ophichthus zophochir*, the analysis resulted in four MOTUs (Figure 4). The first one grouped three individuals from northern Peru. The second MOTU included a larva from the open ocean, the third MOTU comprised one individual from Northern Peru; while the last MOTU grouped the two remaining samples from Northern Peru with two individuals from the coast of California and another larva from the open ocean. Species delimitation of *Malacoctenus tetranemus* resulted in two MOTUs (Figure 5): The first corresponds to the sample PeMar1165 (northern Peru). The second grouped the remaining samples from northern Peru with samples from South Baja California and the Galapagos Archipelago (Isabela Island).

**FIGURE 6:**
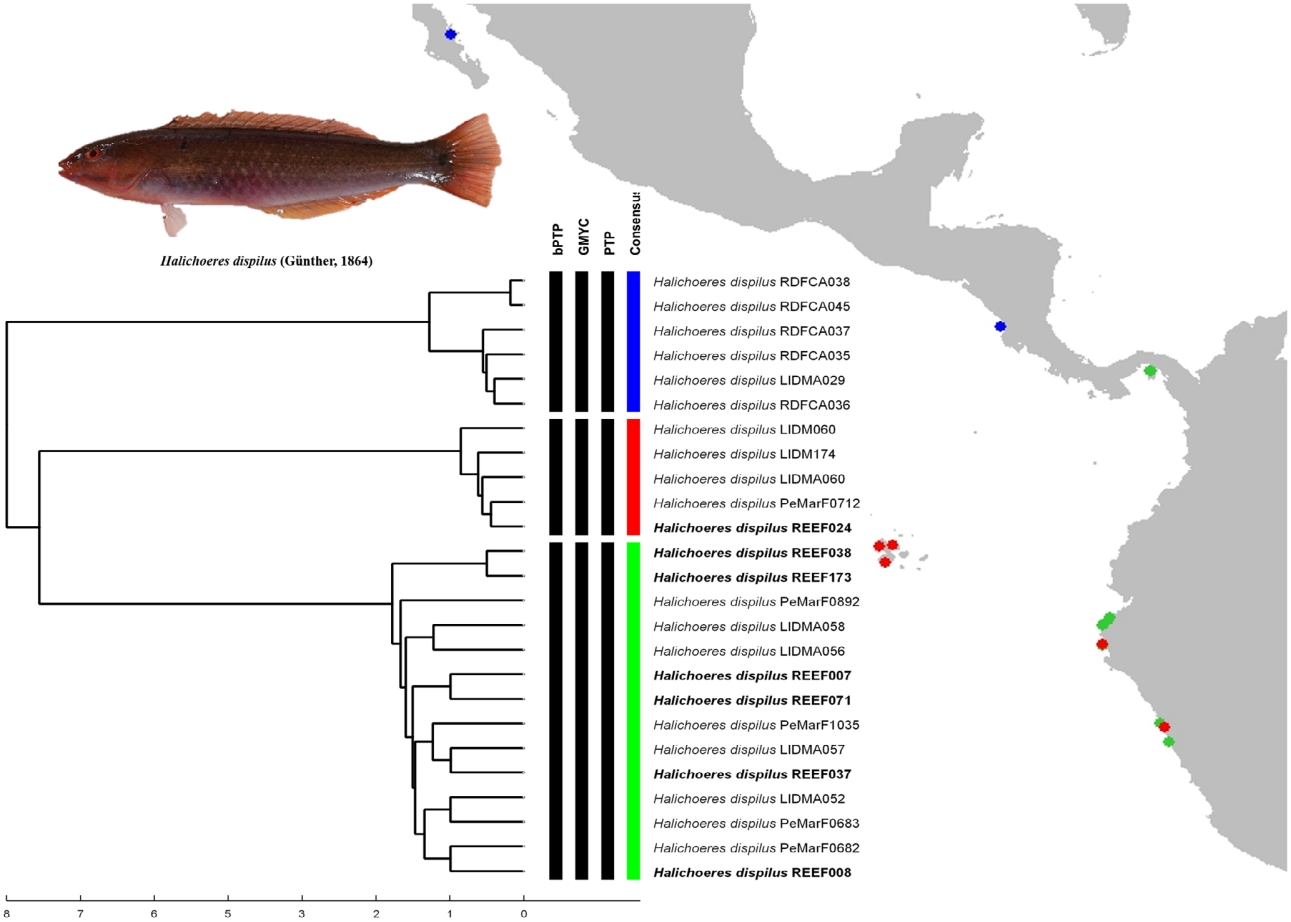
Species delimitation of *H. dispilus* from the EP. Individuals in bold are samples from northern Peru that were used in the main analysis.

Finally, for *H. dispilus*, the delimitation analysis resulted in three MOTUs (Figure 6). The first MOTU clustered individuals from the coast of Costa Rica with one specimen from South Baja California. The second MOTU comprised individuals from the Galapagos archipelago, one specimen from Isla Foca (Northern Peru) and one specimen from Central Peru. In the third MOTU, individuals from Northern and Central Peru were grouped with samples from the Pacific coast of Panama.

## 4 DISCUSSION

Fishes are the biggest vertebrate group, with a notable morphological diversity and biological adaptations that allow the colonization of a variety of habitats and distinction from other species (Nelson et al., 2016). However, the considerable phenotypic plasticity and interspecific superpositions, whether attributed to overlapping meristic data or the presence of similar morphotypes, pose challenges in fish identification when assessing communities with a rich biota, as commonly encountered in tropical reef communities (Allen & Adrim, 2003; Dominici‐Arosemena & Wolff, 2006); where mean temperatures do not fall below of 18°C (even during the cold season) and it is estimated that most of the approximately 10,000 species are spread in (Bone & Moore, 2007).

The northern coast of Peru is home to scarce rocky reef ecosystems located in the Piura and Tumbes departments. However, these ecosystems are inhabited by most of the typical fish families that occur in coral reef ecosystems around the world, including Labridae, Pomacentridae, Serranidae, Blenniidae, Apogonidae and Chaetodontidae (see Bellwood & Wainwright, 2002). This highlights the importance of supporting capacity of these areas and how they contribute to increase the local marine fish biodiversity. In this study, 82 fish species from 37 families were collected. From that, just three species (*Cheilodactylus variegatus*, *Oplegnathus insignis* and *Scartichthys gigas*) correspond to temperate fish species from Central Peru and their presence in our study area would be related when general sea conditions are abnormally cold (Hooker, 2012).

### 4.1 Genetic distance analysis

Genetic distance analyses showed higher interspecific genetic distances than intraspecific distances for all species. The lowest interspecific distance value (but above 2%) was found between samples of *Kyphosus vaigiensis* and *Kyphosus elegans*. The family Kyphosidae corresponds to an important fraction of herbivore species from tropical and temperate reef fish communities (Knudsen & Clements, 2016) and the lack of differentiation of morphological characters has complicated both their identification and the taxonomic evaluation of their species; with different authors (Knudsen & Clements, 2013; Sakai & Nakabo, 2014) presenting contraindications respect the number of valid species, resulting in misidentifications, synonyms and questions about their spatial distribution (Bañón et al., 2017; Valencia, 2016; Valencia et al., 2018). Nevertheless, for this case, external differentiations can be observed between both species (Figure S3a); with a silver brown coloration pattern in *K. elegans* and occasionally, uniform brown mottled with irregularly distributed white spots throughout the body (Figure S3b); which represents an aggressive territorial behavior that this species and *K. sectatrix* can exhibit. (Knudsen & Clements, 2013).

On the other hand, *K. vaigiensis* presents notable golden lines along the body (Figure S3c). Knudsen et al. (2019) showed that this family formed as a result of a recent speciation, colonizing first high latitude regions and then moving to tropical environments during the Miocene from the Indo‐Pacific and finally diversifying in the other regions of the world during the Pliocene; after the closure of the Isthmus of Panama (3.1–3.5 million years); and the Pleistocene, approximately 2.6 million years ago. This scenario could explain the 2.5% interspecific value found between *Kyphosus* species in this study.

### 4.2 Species delimitation

The consensus of the delimitation analysis of the 82 nominal species identified in this study resulted in 87 MOTUs. As *M. cephalus*, *O. zophochir*, *M. tetranemus*, *A. seemanni* and *H. dispilus* showed more than one MOTU, additional analyses were performed in these species.

#### 4.2.1 Mugil cephalus

The species delimitation of *M. cephalus* resulted in the formation of two COI lineages (Figure 3). These correspond to the lineages *Mugil* sp. “A” and *Mugil* sp. “G” lineages, 2 of the 15 mtDNA lineages described by Durand et al. (2012) and Durand and Borsa (2015). This fish is an euryhaline species distributed worldwide that generally inhabits coastal areas (lagoons, bays and estuaries between 42° N and 42° S) and migrates to the open sea to reproduce (Colín et al., 2020; Shen et al., 2011). In general, closely related species with morphological similitudes can result in incongruent taxonomic classification between studies, resulting in a good candidate for genetic analysis of cryptic diversity (Neves et al., 2020). Our results suggest that the *Mugil* sp. “A” lineage corresponds to *M. cephalus* individuals from the TSA realm, while the *Mugil* sp. “G” lineage comprises individuals from the TEP. The occurrence of one individual of the *Mugil* sp. “A” lineage (sample REEF051) in an area of tropical characteristics (Cabo Blanco is the natural limit of tropical fauna) can be explained by two factors. First, the presence of abnormally cold waters during our fieldwork (consistently with the capture of temperate species, see above); could lead to the presence of vagrant temperate fish fauna from the TSA realm above parallel 4° LS. Second, the presence of a transition zone between Cabo Blanco and Punta Aguja (Hooker et al., 2013), allows the presence of both tropical and temperate fauna, instead of acting as a biogeographic barrier (Barahona et al., 2019).

#### 4.2.2 Ophichthus zophochir

For this species, our main analysis resulted in two MOTUs and when analyzed in conjunction with conspecific individuals from the Eastern Pacific, were split into four lineages, three from the TEP and one from Northeastern Pacific, which include two individuals from Northern Peru (in purple, see Figure 4); two areas separated by more than 5000 km.

This benthic species spans the entire tropical area (TEP) from Eureka (California) to Paita (Piura‐Transition zone); with occasional occurrence in Huacho and Callao (Central Peru, perhaps due the ENSO events, Chirichigno & Cornejo, 2001), and extend well into the Northeastern Pacific (McCosker & Rosenblatt, 1998); although the authors remark that these records do not reflect the existence of a self‐sustaining population in the region, as ophichthid leptocephali have never been taken north of Punta San Juanico, Baja California (26°LN). Thus, the last MOTU represents the typical range distribution of this species, and records from California might correspond to unusual occurrences in the temperate region.

The two larvae sampled in the open ocean are an intriguing scenario. One of them was grouped with samples from the fourth MOTU, while the other formed a separate MOTU. Due to their distance from the Galapagos, one might suggest that this individual could be evidence of a separate lineage occurring in these islands. However, *O. zophochir* has never been reported in the Galapagos islands, or any insular area (McCosker & Rosenblatt, 1998). Therefore, the most probable explanation for the larval occurrence in the open ocean is the action of ocean currents that transported drift larvae from Baja California (fourth MOTU, in purple) and other coastal areas inside the TEP that have not been sampled yet. Finally, the third MOTU (in green) could represent a lineage of recent speciation in sympatry with the lineage of northern Peru (first MOTU, in red). Nonetheless, samples from the area between California and Peru are necessary to clarify these relationships.

#### 4.2.3 Malacoctenus tetranemus

In this species, the sample PeMar1165 displayed a genetic divergence of 3.79% and formed a separate MOTU (Figure 5). This result is in agreement with the study done by Pedraza‐Marrón (2014); who found genetic partitions of similar values (3.9%, using Cytb) and suggested that *M. tetranemus* represent a species complex of two lineages: a TEP lineage and a Continental Ecuador lineage; where the last one represents a relict population product of a glaciation during the Pleistocene and now, exists in sympatry with the TEP lineage. In this way, the sample PeMarF1165 represents a member of this Continental Ecuador lineage; while the remaining samples from South Baja California, Huatulco, Galapagos and Cabo Blanco are members of the TEP lineage. Nevertheless, the author did not discard the possibility that this relict lineage of *M. tetranemus* could represent a different species, as both lineages can be vaguely differentiated in coloration pattern.

#### 4.2.4 Ariopsis seemanni

The sample REEF199 showed a genetic differentiation of 3.32% and formed an additional MOTU for *A. seemanni* in our analysis. Although we could not perform a separate analysis, we found two possible explanations for this result. The first explanation could be a misidentification during our morphological analysis, since *Ariopsis simonsi*, a junior synonym of *A. seemanni*, has been recently redescribed as a valid species by Marceniuk et al. (2017) and its range distribution (from Colombia to Talara – Peru, see Figure S2) is more related with our study area. Thus, we could suggest that the sample REEF199 corresponds to a vagrant individual of *A. seemanni* from northern localities and the three remaining catfishes will be *A. simonsi* individuals. (or vice versa). In this case, it would be necessary a taxonomic revision of *Ariopsis* species that occur in northern Peru.

The second is that these catfishes could potentially represent cryptic species. Marceniuk et al. (2017), suggested the existence of a species complex in *A. seemanni* segregated by geography in the TEP; as their molecular analysis failed to support the monophyly of this species. In addition, according to Marceniuk et al. (2023), both low dispersal capacity and high level of endemism of the Ariidae are conditions addressed in studies that describe recent speciation processes related to vicariance events or adaptation to environments with different selective pressure—specifically, a parapatric speciation.

#### 4.2.5 Halichoeres dispilus

The sample REEF024 from Isla Foca displayed a genetic divergence of 2.51% (see Table 1) with the rest of the samples from Northern Peru and in the delimitation analysis, formed a MOTU together with samples from the Galapagos, resulting in three COI lineages (see Figure 6): one of insular and two of continental individuals; a pattern that is concordant with the biogeographic subdivision of the TEP (Cortez, Panama and Oceanic island provinces) proposed by Robertson and Cramer (2009). Furthermore, by comparing genetic distances among locations (See Table S3), we noticed that *H. dispilus* individuals from Panama‐North Peru and Costa Rica‐South Baja California (continental‐continental) displayed shorter genetic distances rather than ones from North Peru‐Galapagos (continental‐insular).

A similar scenario was observed in *Halichoeres* species from Western Atlantic by Rocha et al. (2005). The authors compared mtDNA sequences (Cytochrome b) of 5 wrasses species, using a Mantel test, and found that genetic distances were not related to the geographic distances between different localities and environmental differences would generate a selective pressure between insular and continental specimens among reef fishes from Western Atlantic. In the same way for the TEP, Robertson and Cramer (2009) suggested that the large masses of water that separate the oceanic islands from continental areas would not act as an effective biogeographic barrier and in many cases, ecological differences between and within provinces would be those involved in the formation of endemisms that differentiates insular and continental fauna; resulting again, in parapatric speciation.

Parapatric speciation occurs when new species develop in neighboring but spatially distinct habitats, maintaining areas of contact and continuing some gene flow (Hollocher, 2013); and may be caused by populations adapting to different ecological habitats (Feder et al., 2021). Since *H. dispilu*s displays a long pelagic larval duration (see Victor & Wellington, 2000) that is subjected to a dynamical oceanic circulation (see Montes, 2014; Robertson & Allen, 2015) and phylogeographic breaks can occur within a continuously distributed species even when there are no barriers to gene flow (Irwin, 2002), it is possible to hypothesize that the existence of cryptic species complex in *H. dispilus* are a product of parapatric speciation. However, a comprehensive study including a sampling along the entire distribution together with a multilocus approach is needed to test this hypothesis.

Struck et al. (2018a) emphasize the importance of identify and quantify detailed information about phenotypic disparity to define cryptic species. Although the absence of morphological comparisons doesn't allow us to confirm the occurrence of a species complex in *H. dispilus*, both analysis (Genetic Distances‐Species delimitation) and their relationship with ecological differences (lineages in response to insular and continental ecological conditions) can serve as a first signal of evidence for the hypothesis of the existence of different evolutionary lineages in this species. As De Queiroz (2007) remarks in its ‘Unified species concept’, most of the properties emphasized under the alternative species concepts (such as phenetic distinguishability, reciprocal monophyly, reproductive isolation, etc.) should be considered relevant to the issue of species delimitation; where the presence of one single property constitutes evidence (which can be rejected if interpreted inappropriately) to accept the hypothesis.

Finally, as De Queiroz (2007) suggests, a highly corroborated hypothesis of lineage separation requires multiple lines of evidence and, given that the application of uniparental markers (such as COI) does not provide a comprehensive assessment of gene flow and reproductive isolation, and instead, high genetic divergence in COI can reflects, for example, deep population structure rather than species differences (Struck et al., 2018b), further integrative studies including different molecular markers and approaches are also required to accept or reject our hypothesis of the existence of a cryptic species complex in *H. dispilus* and probably, in many other rocky reef fish species from the TEP.

## AUTHOR CONTRIBUTIONS

**E. Zavala:** Formal analysis (equal); investigation (equal); methodology (equal); visualization (lead); writing – original draft (lead); writing – review and editing (equal). **R. Britzke:** Conceptualization (equal); formal analysis (equal); funding acquisition (equal); investigation (equal); methodology (equal); project administration (equal); resources (equal); supervision (equal); validation (equal); writing – review and editing (equal). **Z. Siccha‐Ramírez:** Conceptualization (equal); formal analysis (equal); funding acquisition (equal); investigation (equal); methodology (equal); project administration (equal); resources (equal); supervision (equal); validation (equal); writing – review and editing (equal). **J. L. Ramirez:** Conceptualization (equal); formal analysis (equal); funding acquisition (equal); investigation (equal); methodology (equal); project administration (equal); resources (equal); supervision (equal); validation (equal); visualization (equal); writing – original draft (supporting); writing – review and editing (equal).

## FUNDING INFORMATION

This research was conducted with the financial support of the National Council of Science, Technology and Technological Innovation (National Fund for Scientific, Technological and Technological Innovation Development–Fondecyt) from Perú. (Grant Number of the project: 363‐2019).

## CONFLICT OF INTEREST STATEMENT

No potential competing interest was reported by the authors.

## Supporting information


Appendix S1

## Data Availability

All data associated with this study are publicly available in GenBank (PP390264 – PP390495) and BOLD systems.

## References

[ece370125-bib-0001] Aburto, O. , & Balart, E. (2001). Community structure of reef fish in several habitats of a rocky reef in the Gulf of California. Marine Ecology, 22(4), 283–305. 10.1046/j.1439-0485.2001.01747.x

[ece370125-bib-0002] Allen, G. R. , & Adrim, M. (2003). Coral reef fishes of Indonesia. Zoological Studies, 42, 1–72.

[ece370125-bib-0004] Bañón, R. , Barros‐García, D. , & de Carlos, A. (2017). Integrative taxonomy supports the presence of two species of *Kyphosus* (Perciformes: Kyphosidae) in Atlantic European waters. Scientia Marina, 81(4), 467–475. 10.3989/scimar.04601.08A

[ece370125-bib-0005] Barahona, S. P. , Vélez‐Zuazo, X. , Santa‐Maria, M. , & Pacheco, A. S. (2019). Phylogeography of the rocky intertidal periwinkle *Echinolittorina paytensis* through a biogeographic transition zone in the southeastern Pacific. Marine Ecology, 40(4), e12556. 10.1111/maec.12556

[ece370125-bib-0006] Bellwood, D. R. , & Wainwright, P. C. (2002). The history and biogeography of fishes on coral reefs. In P. F. Sale (Ed.), Coral reef fishes (pp. 5–32). Elsevier Science. 10.1016/B978-0-12-409548-9.11059-0

[ece370125-bib-0007] Bickford, D. , Lohman, D. J. , Sodhi, N. S. , Ng, P. K. L. , Meier, R. , Winker, K. , Ingram, K. K. , & Das, I. (2007). Cryptic species as a window on diversity and conservation. Trends in Ecology & Evolution, 22(3), 148–155. 10.1016/j.tree.2006.11.004 17129636

[ece370125-bib-0008] Bone, Q. , & Moore, R. H. (2007). Biology of fishes (E. Owen, ed.) (3rd ed.). Taylor & Francis Group.

[ece370125-bib-0009] Bouckaert, R. , Timothy, G. , Vaughan, J. , Barido‐Sottani Fourment, M. , Gavryushkina, A. , Heled, J. , Graham, J. , Kühnert, D. , De Maio, N. , Matschiner, M. , Mendes, F. , Müller, N. , Ogilvie, H. , du Plessis, L. , Popinga, A. , Rambaut, A. , Rasmussen, D. , Siveroni, I. , Suchard, M. , … Drummond, J. (2019). BEAST 2.5: An advanced software platform for Bayesian evolutionary analysis. PLoS Computational Biology, 15(4), e1006650. 30958812 10.1371/journal.pcbi.1006650 PMC6472827

[ece370125-bib-0010] Britzke, R. , Siccha‐Ramirez, Z. R. , & Guevara‐Torres, M. L. (2021). Crossing the borders: First record of bleeding wrasse, Polylepion cruentum Gomon, 1977 (Labriformes, Labridae), in the northern Peru. Papéis Avulsos de Zoologia, 61, e20216185.

[ece370125-bib-0013] Chirichigno, N. , & Cornejo, R. (2001). Catálogo comentado de los peces marinos del Perú. Instituto del Mar del Perú. Publicación especial. Callao – Perú.

[ece370125-bib-0014] Chirichigno, N. , & Vélez, J. (1998). Clave para identificar los peces marinos del Perú. Instituto del Mar del Perú. Publicación Especial, Callao – Perú.

[ece370125-bib-0015] Colín, A. , Hernández‐Pérez, Z. , Guevara‐Chumacero, L. M. , Castañeda‐Rico, S. , Serrato‐Díaz, A. , & Ibáñez, A. L. (2020). Are striped mullet (*Mugil cephalus*) philopatric? Marine Biology, 167(1), 1–15. 10.1007/s00227-019-3622-1

[ece370125-bib-0016] Darriba, D. , Taboada, G. , Doallo, R. , & Posada, D. (2012). jModelTest2: More models, new heuristics and parallel computing. Nature Methods, 9(8), 772. 10.1038/nmeth.2109 PMC4594756 22847109

[ece370125-bib-0017] De Queiroz, K. (2007). Species concepts and species delimitation. Systematic Biology, 56(6), 879–886. 10.1080/10635150701701083 18027281

[ece370125-bib-0018] Dominici‐Arosemena, A. , & Wolff, M. (2006). Reef fish community structure in the tropical eastern Pacific (Panamá): Living on a relatively stable rocky reef environment. Helgoland Marine Research, 60(4), 287–305. 10.1007/s10152-006-0045-4

[ece370125-bib-0019] Durand, J. D. , & Borsa, P. (2015). Mitochondrial phylogeny of grey mullets (Acanthopterygii: Mugilidae) suggests high proportion of cryptic species. Comptes Rendus – Biologies, 338(4), 266–277. 10.1016/j.crvi.2015.01.007 25744223

[ece370125-bib-0020] Durand, J. D. , Shen, K. N. , Chen, W. J. , Jamandre, B. W. , Blel, H. , Diop, K. , Nirchio, M. , Garcia de León, F. J. , Whitfield, A. K. , Chang, C. W. , & Borsa, P. (2012). Systematics of the grey mullets (Teleostei: Mugiliformes: Mugilidae): Molecular phylogenetic evidence challenges two centuries of morphology‐based taxonomy. Molecular Phylogenetics and Evolution, 64(1), 73–92. 10.1016/j.ympev.2012.03.006 22445821

[ece370125-bib-0021] Feder, J. L. , Hood, G. R. , Doellman, M. M. , Schuler, H. , Miller, L. A. , Tait, C. , Glover, M. M. , & Meyers, P. J. (2021). Speciation, process of. In B. D. Roitberg (Ed.), Reference module in life sciences. Elsevier. 10.1016/B978-0-12-822562-2.00025-6

[ece370125-bib-0022] Fujisawa, T. , & Barraclough, T. G. (2013). Delimiting species using single‐locus data and the generalized mixed Yule coalescent approach: A revised method and evaluation on simulated data sets. Systematic Biology, 62(5), 707–724. 10.1093/sysbio/syt033 23681854 PMC3739884

[ece370125-bib-0023] Hall, T. (1999). BioEdit: A user‐friendly biological sequence alignment editor and analysis program for Windows 95/98/NT. Nucleic Acids Symposium Series, 41, 95–98.

[ece370125-bib-0024] Hasegawa, M. , Kishino, H. , & Yano, T. (1985). Dating of the human‐ape splitting by a molecular clock of mitochondrial DNA. Journal of Molecular Evolution, 22(2), 160–174. 10.1007/BF02101694 3934395

[ece370125-bib-0025] Hebert, P. , Stoeckle, M. , Zemlak, T. , & Francis, C. (2004). Identification of birds through DNA barcodes. PLoS Biology, 2(10), e312. 10.1371/journal.pbio.0020312 15455034 PMC518999

[ece370125-bib-0026] Hebert, P. D. N. , Cywinska, A. , Ball, S. L. , & DeWaard, J. R. (2003). Biological identifications through DNA barcodes. Proceedings of the Royal Society B: Biological Sciences, 270(1512), 313–321. 10.1098/rspb.2002.2218 PMC1691236 12614582

[ece370125-bib-0027] Hollocher, H. (2013). Speciation, theories of. In S. A. Levin (Ed.), Encyclopedia of biodiversity: Second edition (Vol. 6, pp. 629–639). Elsevier. 10.1016/B978-0-12-384719-5.00130-1

[ece370125-bib-0084] Hooker, Y. (1990). Primer Registro de Johnrandallia nigrirostris, Gill 1862, en el Perú. Boletin de Lima. Nº, 68, 69–71.

[ece370125-bib-0085] Hooker, Y. (1993). Zonación de los peces del Litoral Rocoso en el Área Comprendida entre las Localidaes de Cabo Blanco y Los Órganos. [Thesis to opt for the degree of Bachellor in Biological Sciences]. Universidad Nacional De Trujillo.

[ece370125-bib-0086] Hooker, Y. (2000). Microspathodon dorsalis (Pisces: Pomacentridae) y Prionurus laticlavius (Pisces: Acanthuridae), dos nuevos registros para el mar peruano. Inf. Prog. Mar del Perú. Nº 117.

[ece370125-bib-0028] Hooker, Y. (2009). Nuevos registros de peces costeros tropicales para el Perú. Revista Peruana de Biología, 16(1), 33–41.

[ece370125-bib-0029] Hooker, Y. (2012). Evaluación de la Biodiversidad submarina del norte de Piura (Cabo Blanco – Los Órganos). Informe de primera etapa. Universidad Peruana Cayetano Heredia. Naturaleza y Cultura Internacional y Gobierno Regional de Piura.

[ece370125-bib-0030] Hooker, Y. , Prieto‐Rios, E. , & Solís‐Marin, F. A. (2013). Echinoderms of Peru. In J. J. Alvarado & F. A. Solis‐Marin (Eds.), Echinoderm research and diversity in Latin America (pp. 277–299). Springer.

[ece370125-bib-0031] Hooker, Y. , & Ubillus, O. (2011). Propuesta de creación de la Reserva Nacional El Ñuro y Arrecifes de Punta Sal . 1–60.

[ece370125-bib-0033] Hubert, N. , Meyer, C. P. , Bruggemann, H. J. , Guérin, F. , Komeno, R. J. L. , Espiau, B. , Causse, R. , Williams, J. T. , & Planes, S. (2012). Cryptic diversity in indo‐pacific coral‐reef fishes revealed by DNA‐barcoding provides new support to the centre‐of‐overlap hypothesis. PLoS One, 7(3), e28987. 10.1371/journal.pone.0028987 22438862 PMC3305298

[ece370125-bib-0034] Irwin, D. E. (2002). Phylogeographic breaks without geographic barriers to gene flow. Evolution, 56(12), 2383–2394. 10.1111/j.0014-3820.2002.tb00164.x 12583579

[ece370125-bib-0035] Jacobina, U. , Torres, R. , De Mello Affonso, P. , Dos Santos, E. , Calado, L. , & De Araújo Bitencourt, J. (2020). DNA barcoding reveals cryptic diversity and peculiar phylogeographic patterns in mojarras (Perciformes: Gerreidae) from the Caribbean and South‐western Atlantic. Journal of the Marine Biological Association of the United Kingdom, 100(2), 277–283. 10.1017/S0025315419001206

[ece370125-bib-0036] Jiménez, S. (1999). Abundancia y estructura comunitaria de peces de arrecife rocoso en la zona de Isla Cerralvo, B.C.S., México [Thesis to opt for the degree of Master of Science]. Interdisciplinary Center for Marine Sciences.

[ece370125-bib-0037] Kimura, M. (1980). A simple method for estimating evolutionary rates of base substitutions through comparative studies of nucleotide sequences. Journal of Molecular Evolution, 16, 111–120. 10.1007/BF01731581 7463489

[ece370125-bib-0038] Knudsen, S. , Choat, J. , & Clements, K. (2019). The herbivorous fish family Kyphosidae (Teleostei: Perciformes) represents a recent radiation from higher latitudes. Journal of Biogeography, 46(9), 2067–2080. 10.1111/jbi.13634

[ece370125-bib-0039] Knudsen, S. , & Clements, K. (2013). Revision of the fish family Kyphosidae. Zootaxa, 3751(1), 1–101. 10.11646/zootaxa.3751.1 29097648

[ece370125-bib-0040] Knudsen, S. , & Clements, K. (2016). World‐wide species distributions in the family Kyphosidae (Teleostei: Perciformes). Molecular Phylogenetics and Evolution, 101, 252–266. 10.1016/j.ympev.2016.04.037 27143240

[ece370125-bib-0041] Kumar, S. , Stecher, G. , Li, M. , Knyaz, C. , & Tamura, K. (2018). MEGA X: Molecular evolutionary genetics analysis across computing platforms. Molecular Biology and Evolution, 35, 1547–1549. 10.1093/molbev/msy096 29722887 PMC5967553

[ece370125-bib-0042] Lambarri, M. , & Espinosa, H. (2018). Métodos de colecta y preservación de peces: Manual de campo y laboratorio. Universidad Nacional Autónoma de México.

[ece370125-bib-0044] Marceniuk, A. P. , Acero, A. P. , Cooke, R. , & Betancur‐R, R. (2017). Taxonomic revision of the New World genus *Ariopsis* Gill (Siluriformes: Ariidae), with description of two new species. Zootaxa, 4290(1), 1–42. 10.11646/zootaxa.4290.1.1

[ece370125-bib-0045] Marceniuk, A. P. , Oliveira, C. , & Ferraris, C. J. (2023). A new classification of the family Ariidae (Osteichthyes: Ostariophysi: Siluriformes) based on combined analyses of morphological and molecular data. Zoological Journal of the Linnean Society, XX, 1–51. 10.1093/zoolinnean/zlad078

[ece370125-bib-0046] McCosker, J. E. , & Rosenblatt, R. H. (1998). A revision of the eastern Pacific Snake‐eel genus Ophichthus (Anguilliformes: Ophichthidae) with the description of six new species. Proceedings of the California Academy of Sciences, 50(19), 397–432.

[ece370125-bib-0047] Miller, M. A. , Pfeiffer, W. , & Schwartz, T. (2010). Creating the CIPRES Science Gateway for inference of large phylogenetic trees . 2010 Gateway Computing Environments Workshop, GCE 2010. 10.1109/GCE.2010.5676129

[ece370125-bib-0048] Montes, I. (2014). La circulación del Pacífico tropical este y su conexión con el Peru. Boletín técnico: Generación de Modelos climáticos Para el pronóstico de la Ocurrencia del Fenómeno El Niño, 1(4), 4–7.

[ece370125-bib-0049] Msangameno, D. J. (2016). Intertidal and Nearshore Rocky Reefs. In United Nations Environment Programme (Ed.), Regional state of the coast report (pp. 81–98). Institute of Marine Sciences. 10.18356/dd8dca69-en

[ece370125-bib-0149] Nelson, J. S. , Grande, T. C. , & Wilson, M. V. C. (2016). Fishes of the World (5th edn). John Wiley & Sons, Inc.

[ece370125-bib-0050] Neves, J. M. M. , Almeida, J. P. F. A. , Sturaro, M. J. , FabrÉ, N. N. , Pereira, R. J. , & Mott, T. (2020). Deep genetic divergence and paraphyly in cryptic species of Mugil fishes (Actinopterygii: Mugilidae). Systematics and Biodiversity, 18(2), 116–128. 10.1080/14772000.2020.1729892

[ece370125-bib-0051] Palmerín‐Serrano, P. N. , Tavera, J. , Espinoza, E. , Angulo, A. , Martínez‐Gómez, J. E. , González‐Acosta, A. F. , & Domínguez‐Domínguez, O. (2021). Evolutionary history of the reef fish *Anisotremus interruptus* (Perciformes: Haemulidae) throughout the tropical eastern Pacific. Journal of Zoological Systematics and Evolutionary Research, 59(1), 148–162. 10.1111/jzs.12392

[ece370125-bib-0052] Pedraza‐Marrón, C. (2014). Historia evolutiva de género Malacoctenus (Labrisomidae) en el Pacifico oriental tropical. [Thesis to opt for the degree of Master of Biological Sciences]. Universidad Michoacana de San Nicolás de Hidalgo.

[ece370125-bib-0083] Rambaut, A. , & Drummond, J. (2009). FigTree version 1.4.3 . http://tree.bio.ed.ac.uk/software/figtree/

[ece370125-bib-0053] Rambaut, A. , Drummond, A. , Xie, D. , Baele, G. , & Suchard, M. (2018). Posterior summarisation in Bayesian phylogenetics using tracer 1.7. Systematic Biology, 67, 901–904. 29718447 10.1093/sysbio/syy032 PMC6101584

[ece370125-bib-0055] Ramirez, J. L. , Valdivia, P. , Rosas‐Puchuri, U. , & Valdivia, N. L. (2023). SPdel: A pipeline to compare and visualize species delimitation methods for single‐locus datasets. Molecular Ecology Resources, 23(8), 1959–1965. 10.1111/1755-0998.13864 37702121

[ece370125-bib-0057] Ratnasingham, S. , & Hebert, P. (2007). BOLD: The barcode of life data system (www.barcodinglife.org). Molecular Ecology Notes, 7, 355–364. 10.1111/j.1471-8286.2007.01678.x 18784790 PMC1890991

[ece370125-bib-0059] Robertson, R. , & Allen, G. (2015). Peces Costeros del Pacífico Oriental Tropical: Sistema de Información en línea. Versión 2.0. Instituto Smithsonian de Investigaciones Tropicales.

[ece370125-bib-0060] Robertson, R. , & Cramer, K. (2009). Shore fishes and biogeographic subdivisions of the tropical eastern Pacific. Marine Ecology Progress Series, 380, 1–17. 10.3354/meps07925

[ece370125-bib-0061] Rocha, L. A. , & Bowen, B. (2008). Speciation in coral‐reef fishes. Journal of Fish Biology, 72, 1101–1121.

[ece370125-bib-0062] Rocha, L. A. , Robertson, D. R. , Roman, J. , & Bowen, B. W. (2005). Ecological speciation in tropical reef fishes. Proceedings of the Royal Society B: Biological Sciences, 272(1563), 573–579. 10.1098/2004.3005 PMC1564072 15817431

[ece370125-bib-0087] Sakai, K. , & Nakabo, T. (2014). Taxonomic review of Kyphosus (*Pisces: Kyphosidae*) in the Atlantic and Eastern Pacific Oceans. Ichthyological Research, 61(3), 265–292. 10.1007/s10228-014-0395-x

[ece370125-bib-0063] Sanchez, A. , Aburto, O. , Erisman, B. , Jiménez, V. , & Hinojosa, G. (2015). Rocky reefs: Preserving biodiversity for the benefit of the communities in the aquarium of the world . 10.1007/978-3-319-23763-3_11

[ece370125-bib-0064] Sarhan, M. , Azab, A. , Khalaf‐Allah, H. , & Afifi, M. (2019). DNA barcoding supports sexual dimorphism in two Labrid species; *Cheilinus lunulatus* and *Halichoeres hortulanus* (family Labridae) in Red Sea, Egypt. The Egyptian Journal of Aquatic Research, 45(4), 395–401. 10.1016/j.ejar.2019.10.002

[ece370125-bib-0065] SERNANP . (2018). Mar tropical de Grau National Reservoir: Final expedient .

[ece370125-bib-0066] Shen, K. N. , Jamandre, B. W. , Hsu, C. C. , Tzeng, W. N. , & Durand, J. D. (2011). Plio‐Pleistocene sea level and temperature fluctuations in the northwestern Pacific promoted speciation in the globally‐distributed flathead mullet Mugil cephalus. BMC Evolutionary Biology, 11(1), 1–17. 10.1186/1471-2148-11-83 21450111 PMC3079632

[ece370125-bib-0067] Siccha‐Ramirez, R. , Luque, C. , Vera, M. , Britzke, R. , Guevara, M. , Castillo, D. , & Miranda, J. (2022). Catálogo ilustrado de la ictiofauna de la región Tumbes (p. 490). Instituto del Mar del Peru.

[ece370125-bib-0069] Spalding, M. , Fox, H. , Allen, G. , Davidson, N. , Ferdaña, Z. , Finlayson, M. , Halpern, B. , Jorge, M. , Lombana, A. , Lourie, S. , Martin, K. , McManus, E. , Molnar, J. , Recchia, C. , & Robertson, J. (2007). Marine ecoregions of the world: A bioregionalization of coast and shelf areas. Bioscience, 57(7), 573–583.

[ece370125-bib-0071] Struck, T. H. , Feder, J. L. , Bendiksby, M. , Birkeland, S. , Cerca, J. , Gusarov, V. I. , Kistenich, S. , Larsson, K. H. , Liow, L. H. , Nowak, M. D. , Stedje, B. , Bachmann, L. , & Dimitrov, D. (2018a). Finding evolutionary processes hidden in cryptic species. Trends in Ecology & Evolution, 33(3), 153–163. 10.1016/j.tree.2017.11.007 29241941

[ece370125-bib-0072] Struck, T. H. , Feder, J. L. , Bendiksby, M. , Birkeland, S. , Cerca, J. , Gusarov, V. I. , Kistenich, S. , Larsson, K. H. , Liow, L. H. , Nowak, M. D. , Stedje, B. , Bachmann, L. , & Dimitrov, D. (2018b). Cryptic species – more than terminological chaos: A reply to Heethoff. Trends in Ecology & Evolution, 33(5), 310–312. 10.1016/j.tree.2018.02.008 29588071

[ece370125-bib-0073] Tarazona, J. , Gutierrez, D. , & Indacochea, A. (2003). Overview and challenges of marine biodiversity research in Peru. Gayana, 67(2), 206–231.

[ece370125-bib-0074] Thompson, J. D. , Gibson, T. J. , Plewniak, F. , Jeanmougin, F. , & Higgins, D. G. (1997). The CLUSTAL_X windows interface: Flexible strategies for multiple sequence alignment aided by quality analysis tools. Nucleic Acids Research, 25, 4876–4882. 9396791 10.1093/nar/25.24.4876 PMC147148

[ece370125-bib-0075] Valencia, E. (2016). Conectividad entre las Isla de Pascua y la Isla Salas y Gómez: Diferenciación genético poblacional del pez de importancia cultural Kyphosus sandwicensis (Sauvage, 1880). [Thesis to opt for the degree of Master in Biological Sciences]. Universidad de Chile.

[ece370125-bib-0076] Valencia, O. , Domínguez, O. , López, A. , Martínez, J. , & Ayala, A. (2018). Partial albinism in the Revillagigedo sea chub *Kyphosus sectatrix* (Perciformes: Kyphosidae) from Clarion Island, Mexico. Revista Mexicana de Biodiversidad, 89(2), 572–575. 10.22201/ib.20078706e.2018.2.2242

[ece370125-bib-0077] Victor, B. , & Wellington, G. (2000). Endemism and the pelagic larval duration of reef fishes in the eastern Pacific Ocean. Marine Ecology Progress Series, 205, 241–248. 10.3354/meps20524

[ece370125-bib-0078] Vildoso, A. , Vélez, J. , Chirichigno, N. , & Chirinos, A. (1999). Diversidad de peces marinos del Perú. Boletin Instituto del Mar del Perú, 18(1–2), 49–76.

[ece370125-bib-0079] Ward, R. D. , Zemlak, T. S. , Innes, B. H. , Last, P. R. , & Hebert, P. D. N. (2005). DNA barcoding Australia's fish species. Philosophical Transactions of the Royal Society, B: Biological Sciences, 360(1462), 1847–1857. 10.1098/rstb.2005.1716 16214743 PMC1609232

[ece370125-bib-0080] Westneat, M. W. , & Alfaro, M. E. (2005). Phylogenetic relationships and evolutionary history of the reef fish family Labridae. Molecular Phylogenetics and Evolution, 36(2), 370–390. 10.1016/j.ympev.2005.02.001 15955516

[ece370125-bib-0081] Willenz, P. , Hooker, Y. , Hajdu, E. , & Desqueyroux, R. (2010). Taxonomía y Biogeografía de la Fauna de Esponjas del Mar del Perú: Proyecto ESPER . Libro de Resúmenes, II Congreso de Ciencias del Mar del Perú. Piura.

[ece370125-bib-0082] Zhang, J. , Kapli, P. , Pavlidis, P. , & Stamatakis, A. (2013). A general species delimitation method with applications to phylogenetic placements. Bioinformatics, 29(22), 2869–2876. 10.1093/bioinformatics/btt499 23990417 PMC3810850

