# Supplementary material for: DNA barcoding of marine rocky reef fishes from northern Peru suggests a parapatric speciation in the Tropical Eastern Pacific
Source: Ecol Evol. 2025 Mar 5;15(3):e70125. doi: 10.1002/ece3.70125 (PMC11883181; doi:10.1002/ece3.70125)
Supplement: Supplementary file 1 — Appendix S1 [file ECE3-15-e70125-s001.docx]

**Table S1. Fish families collected in the rocky reefs from the Tropical sea of Peru (2018 – 2021) *sensu* Fricke & Van der Laan (2023). Occurrence in our study (TEP: Tropical Eastern Pacific, TZ: Transition Zone, TSA: Temperate South America)**

| Order | Family | Species | Occurrence |
| --- | --- | --- | --- |
| Perciformes | Serranidae | *Alphestes immaculatus* Breder 1936 | TEP |
|  |  | *Alphestes multiguttatus* (Günther, 1867) | TEP |
|  |  | *Cratinus agassizii* Steindachner, 1878 | TEP |
|  |  | *Paralabrax callaensis* Starks, 1906 | TZ |
|  |  | *Serranus huascarii* Steindachner, 1900 | TZ |
|  |  | *Serranus psittacinus* Valenciennes, 1846 | TZ, TEP |
|  |  | *Diplectrum conceptione* (Valenciennes, 1828) | TEP |
|  | Epinephelidae | *Cephalopholis panamensis* (Steindachner, 1876) | TEP |
|  |  | *Mycteroperca xenarcha* Jordan, 1888 | TEP |
|  |  | *Epinephelus labriformis*(Jenyns, 1840) | TEP |
|  |  | *Paranthias colonus* (Valenciennes, 1846) | TEP |
|  |  | *Hyporthodus acanthistius* (Gilbert, 1892) | TEP |
|  | Anthiadidae | *Pronotogrammus multifasciatus* Gill, 1863 | TZ |
|  | Grammistidae | *Rypticus nigripinni*s Gill, 1861 | TEP |
|  | Scorpaenidae | *Scorpaena mystes*Jordan & Starks, 1895 | TZ, TEP |
|  |  | *Scorpaena histrio* Jenyns, 1840 | TZ |
|  |  | *Scorpaenodes xyris* (Jordan & Gilbert, 1882) | TEP |
|  | Triglidae | *Prionotus stephanophrys* Lockington, 1881 | TEP |
| Labriformes | Labridae | *Halichoeres dispilus*(Günther, 1864) | TEP. TZ, TSA |
|  |  | *Halichoeres notospilus*(Günther, 1864) | TEP |
|  |  | *Bodianus diplotaenia*(Gill, 1862) | TEP |
|  |  | *Thalassoma lucasanum*(Gill, 1862) | TEP |
|  |  | *Decodon melasma*Gomon, 1974 | TZ |
|  |  | *Nicholsina denticulata (Evermann & Radcliffe, 1917)* | TEP |
| Centrarchiformes | Latridae | *Chirodactylus variegatus (Valenciennes, 1833)******** | TZ, TEP |
|  | Oplegnathidae | *Oplegnathus insignis*(Kner, 1867)***** | TZ |
|  | Cirrhitidae | *Cirrhitus rivulatus* Valenciennes, 1846 | TEP |
|  | Kyphosidae | *Kyphosus elegans* (Peters, 1869) | TEP |
|  |  | *Kyphosus vaigiensis* (Quoy & Gaimard, 1825) | TEP |
| Siluriformes | Ariidae | *Ariopsis seemanni*(Günther, 1864) | TEP |
| Mugiliformes | Mugilidae | *Mugil cephalus* Linnaeus, 1758 | TEP |
|  |  | *Mugil setosus* Gilbert, 1892 | TEP |
| Acanthuriformes | Chaetodontidae | *Chaetodon humeralis* Günther, 1860 | TEP |
|  |  | *Johnrandallia nigrirostris* (Gill, 1862) | TZ, TEP |
|  | Pomacanthidae | *Pomacanthus zonipectus*(Gill, 1862) | TEP |
|  |  | *Holacanthus passer* Valenciennes, 1846 | TEP |
|  | Ephippidae | *Chaetodipterus zonatus* (Girard, 1858) | TEP |
|  | Lutjanidae | *Lutjanus argentiventris* (Peters, 1869) | TEP |
|  | Sparidae | *Calamus brachysomus* (Lockington, 1880) | TEP |
|  | Haemulidae | *Anisotremus interruptus* (Gill, 1862) | TEP |
| Acanthuriformes | Haemulidae | *Microlepidotus brevipinnis*(Steindachner, 1869) | TEP |
|  | Gerreidae | *Gerres simillimus*Regan, 1907 | TEP |
|  | Sciaenidae | *Pareques lanfeari* (Barton, 1947) | TZ |
|  |  | *Pareques viola* (Gilbert, 1898) | TEP |
|  | Latilidae | *Caulolatilus affinis*Gill, 1865 | TEP |
| Blenniformes | Blenniidae | *Plagiotremus azaleus* (Jordan & Bollman, 1890) | TEP |
|  |  | *Scartichthys gigas*(Steindachner, 1876)* | TZ |
|  |  | *Ophioblennius steindachneri* Jordan & Evermann, 1898 | TEP |
|  |  | *Hypsoblennius paytensis* (Steindachner, 1876) | TEP |
|  |  | *Hypsoblennius brevipinnis* (Günther, 1861) | TEP |
|  | Labrisomidae | *Labrisomus multiporosus* Hubbs, 1953 | TEP |
|  |  | *Malacoctenus tetranemus* (Cope, 1877) | TEP |
| Anguilliformes | Muraenidae | *Gymnothorax phalarus* Bussing, 1998 | TEP |
|  |  | *Gymnothorax equatorialis* (Hildebrand, 1946) | TEP |
|  | Ophichthidae | *Ophichthus zophochir* Jordan & Gilbert, 1882 | TEP |
|  |  | *Echiophis brunneus*(Castro-Aguirre & Suárez de los Cobos, 1983) | TEP |
| Ophidiiformes | Ophidiidae | *Brotula clarkae*Hubbs, 1944 | TEP |
| Tetraodontiformes | Balistidae | *Balistes polylepis* Steindachner, 1876 | TEP |
|  |  | *Pseudobalistes naufragium* (Jordan & Starks, 1895) | TEP |
|  | Diodontidae | *Diodon hystrix* Linnaeus, 1758 | TEP |
|  |  | *Diodon holocanthus* Linnaeus, 1758 | TEP |
|  | Tetraodontidae | *Sphoeroides lobatus* (Steindachner, 1870) | TEP |
|  |  | *Sphoeroides annulatus* (Jenyns, 1842) | TEP |
| Kurtiformes | Apogonidae | *Apogon dovii*Günther, 1862 | TEP |
|  |  | Apogon pacificus (Herre, 1935) | TEP |
| Gobiiformes | Gobiidae | C*oryphopterus urospilus* Ginsburg, 1938 | TEP |
|  |  | *Bathygobius ramosus*Ginsburg, 1947 | TEP |
|  |  | *Lythrypnus dalli* (Gilbert, 1890) | TEP |
| Gobiesociformes | Gobiesocidae | *Tomicodon chilensis* Brisout de Barneville, 1846 | TZ |
| Myliobatiformes | Urotrygonidae | *Urotrygon chilensis* (Günther, 1872) | TEP |
|  |  | *Urobatis tumbesensis* (Chirichigno F. & McEachran, 1979) | TEP |
| Rajiformes | Arhynchobatidae | *Sympterygia brevicaudata* (Cope, 1877) | TZ |
| Carangiformes | Centropomidae | *Centropomus nigrescens*Günther, 1864 | TEP |
| Cichliformes | Pomacentridae | *Azurina atrilobata*(Gill, 1862) | TEP |
|  |  | *Azurina intercrusma* (Evermann & Radcliffe, 1917) | TEP |
|  |  | *Abudefduf troschelii* (Gill, 1862) | TEP |
|  |  | *Abudefduf concolor* (Gill, 1862) | TEP |
|  |  | *Chromis alta* Greenfield & Woods, 1980 | TEP |
|  |  | *Stegastes acapulcoensis* (Fowler, 1944) | TEP |
|  |  | *Stegastes flavilatus*(Gill, 1862) | TEP |
|  |  | *Stegastes rectifraenum*(Gill, 1862) | TEP |
|  |  | *Stegastes beebei*(Nichols, 1924) | TEP |

**Table S2. COI sequences used in the Species delimitation analysis of *O. zophochir, M. cephalus. M. tetranemus* and *H. dispilus*.**

| Species | Country | Sampling location | GenBank accession code | BOLD systems code | Reference |
| --- | --- | --- | --- | --- | --- |
| *Ophichthus zophochir* | - | Pacific Ocean | - | LIDMA1022 | - |
| *Ophichthus zophochir* | - | Pacific Ocean | - | LIDMA1403 | - |
| *Ophichthus zophochir* | EE.UU | California | EU520650 | GBGC5911-08 | - |
| *Ophichthus zophochir* | EE.UU | California - Shellmaker Island | GU440436 | MFC132-08 | - |
| *Mugil cephalus* | Chile | Coast of Iquique | JQ060563 | - | Durand et al. (2012) |
| *Mugil cephalus* | Chile | Coast of Iquique | JQ060564 | - | Durand et al. (2012) |
| *Mugil cephalus* | Mexico | Paredon - Chiapas | JQ060561 | - | Durand et al. (2012) |
| *Mugil cephalus* | Mexico | Paredon - Chiapas | JQ060560 | - | Durand et al. (2012) |
| *Mugil cephalus* | Mexico | Colima - Cuyutlán Lagoon | MN505186 | - | Neves et al. (2020) |
| *Mugil cephalus* | Mexico | Colima - Cuyutlán Lagoon | MN505187 | - | Neves et al. (2020) |
| *Mugil cephalus* | Mexico | Colima - Cuyutlán Lagoon | MN338990 | - | Colin et al. (2020) |
| *Mugil cephalus* | Mexico | Mazatlán | MN338992 | - | Colin et al. (2020) |
| *Mugil cephalus* | Mexico | Mazatlán | MN338993 | - | Colin et al. (2020) |
| *Mugil cephalus* | Mexico | Colima - Cuyutlán Lagoon | MN338994 | - | Colin et al. (2020) |
| *Mugil cephalus* | Mexico | Mazatlán | MN338991 | - | Colin et al. (2020) |
| *Mugil cephalus* | Mexico | La Paz | HQ149715 | - | Durand et al. (2012) |
| *Malacoctenus tetranemus* | Mexico | South Baja California - Tecolote Beach | - | LIDM1373 | - |
| *Malacoctenus tetranemus* | Mexico | Galapagos - Isabela Island | - | LIDM1374 | - |
| *Malacoctenus tetranemus* | Mexico | Galapagos - Isabela Island | - | LIDM1375 | - |
| *Malacoctenus tetranemus* | Mexico | South Baja California - San Lucas cape | - | LIDMA474 | - |
| *Malacoctenus tetranemus* | Ecuador | Galapagos - Isabela Island | - | LIDMA646 | - |
| *Malacoctenus tetranemus* | Ecuador | Galapagos - Isabela Island | - | LIDMA644 | - |
| *Malacoctenus tetranemus* | México | Huatulco | HQ168605 | ANGBF32474-19 | Lin & Hastings (2013) |
| *Halichoeres dispilus* | Ecuador | Galapagos - Isabela Island | - | LIDM060 | - |
| *Halichoeres dispilus* | Ecuador | Galapagos - Santiago Island | - | LIDM174 | - |
| *Halichoeres dispilus* | Ecuador | Galapagos - Isla Island | - | LIDMA060 | - |
| *Halichoeres dispilus* | Costa Rica | Guanacaste - San Jose Island | - | RDFCA038 | - |
| *Halichoeres dispilus* | Costa Rica | Guanacaste - San Jose Island | - | RDFCA035 | - |
| *Halichoeres dispilus* | Costa Rica | Guanacaste - San Jose Island | - | RDFCA037 | - |
| *Halichoeres dispilus* | Costa Rica | Guanacaste - San Jose Island | - | RDFCA036 | - |
| *Halichoeres dispilus* | Costa Rica | Guanacaste - San Jose Island | - | RDFCA045 | - |
| *Halichoeres dispilus* | Panamá | Panama Bay - Contadora Island | - | LIDMA029 | - |
| *Halichoeres dispilus* | Panamá | Panama Bay - Contadora Island | - | LIDMA056 | - |
| *Halichoeres dispilus* | Panamá | Panama Bay - Contadora Island | - | LIDMA058 | - |
| *Halichoeres dispilus* | Panamá | Panama Bay - Contadora Island | - | LIDMA057 | - |
| *Halichoeres dispilus* | Panamá | Panama Bay - Contadora Island | - | LIDMA052 | - |

**Table S3. Between group mean K2P distance (%) calculated for *H. dispilus* individuals using MEGA X. (Groups were defined according the origin of each sample with exception of the samples REEF024 and PeMarF0712 that were included in the Galapagos group as suggested by the delimitation analyses).**

|  | Galápagos | South Baja California | Panamá | Central Perú | Northern Perú | Transition zone | Costa Rica |
| --- | --- | --- | --- | --- | --- | --- | --- |
| Galápagos | 0 |  |  |  |  |  |  |
| South Baja California | 3.174 |  |  |  |  |  |  |
| Panamá | 2.618 | 2.028 |  |  |  |  |  |
| Central Perú | 2.617 | 2.106 | 0.154 |  |  |  |  |
| Northern Perú | 2.569 | 2.059 | 0.223 | 0.231 |  |  |  |
| Transition zone | 2.51 | 2.053 | 0.102 | 0.102 | 0.159 |  |  |
| Costa Rica | 3.173 | 0.185 | 2.091 | 2.169 | 2.121 | 2.116 | 0 |

**Figure S1. Bayesian Inference Tree showing the clustering of MOTUs obtained by the species delimitation of Rocky reef fishes from Northern Peru**

**Figure S2. Bayesian Inference Tree showing the clustering of MOTUs obtained by the species delimitation of Rocky reef fishes from Northern Peru (cont.)**

**Figure S3. Samples of *Kyphosus elegans* with (A) usual coloration and (B), aggressive territorial coloration. (C) *Kyphosus vaigiensis*.**

**

**

**A**





**C**

**B**

**C**

**Figure S4: Range distribution of *A. seemanni* (in red) and *A. simonsi* (in green) according Marceniuk et al. (2017)**


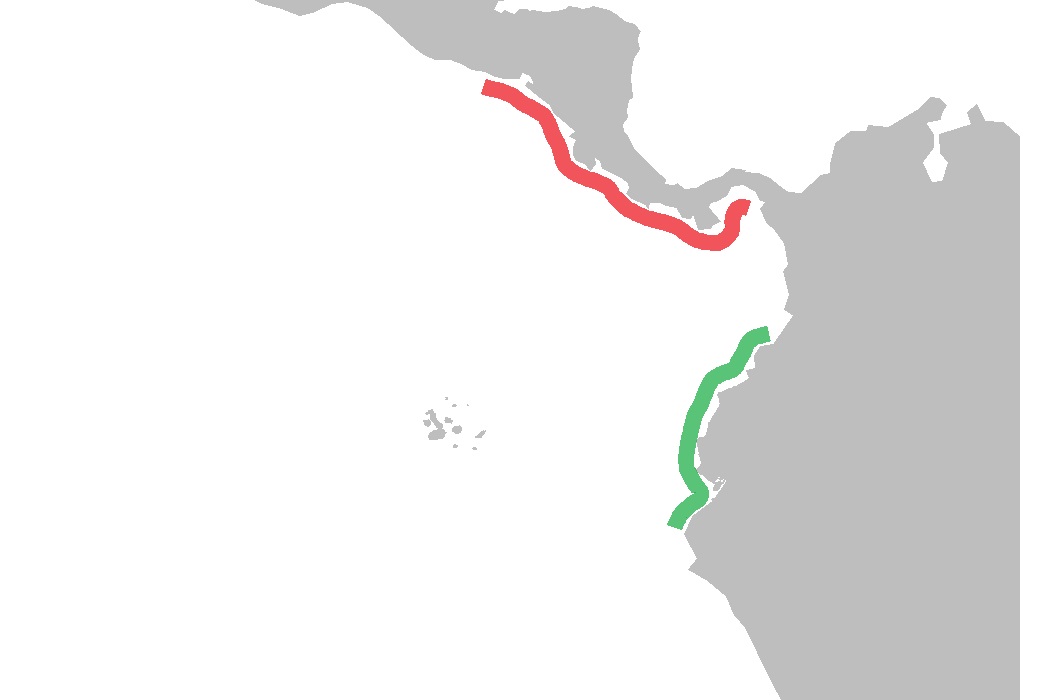


***Ariopsis seemanni* (Günther, 1864)**

***Ariopsis simonsi* (Starks, 1906)**
